# Analysis of spatio-temporal fungal growth dynamics under different environmental conditions

**DOI:** 10.1186/s43008-019-0009-3

**Published:** 2019-06-21

**Authors:** Liselotte De Ligne, Guillermo Vidal-Diez de Ulzurrun, Jan M. Baetens, Jan Van den Bulcke, Joris Van Acker, Bernard De Baets

**Affiliations:** 1 KERMIT, Department of Data Analysis and Mathematical Modelling, Ghent University, Coupure links 653, 9000 Ghent, Belgium; 2 UGent-Woodlab - Laboratory of Wood Technology, Department of Environment, Ghent University, Coupure links 653, 9000 Ghent, Belgium

**Keywords:** Time series analysis, Temperature, Relative humidity, Fungal networks, *Coniophora puteana*, *Rhizoctonia solani*

## Abstract

Traditionally, fungal growth dynamics were assessed manually, limiting the research to a few environmental conditions and/or fungal species. Fortunately, more automated ways of measurement are gaining momentum due to the availability of cheap imaging and processing equipment and the development of dedicated image analysis algorithms. In this paper, we use image analysis to assess the impact of environmental conditions on the growth dynamics of two economically important fungal species, *Coniophora puteana* and *Rhizoctonia solani*. Sixteen environmental conditions combining four temperatures (15, 20, 25 and 30 °C) and four relative humidity (RH) conditions (65, 70, 75 and 80% RH) were tested. Fungal growth characteristics were extracted from images of the growing fungi, taken at regular points in time. Advanced time series analysis was applied to quantitatively compare the effect of the environmental conditions on these growth characteristics. The evolution of the mycelial area and the number of tips over time resulted in typical sigmoidal growth curves. Other growth characteristics such as the mean hyphal segment length did not vary significantly over time. Temperature and RH usually had a combined effect on the growth dynamics of the mycelial area and the number of tips. When defining optimal growth conditions for a fungus, it is therefore of primordial importance that the effect of temperature and RH is assessed simultaneously. At the most extreme conditions we tested, the mycelium most probably experienced water stress when developing over the inert Petri dish surface. An RH of 65% (independent of temperature) for *C. puteana* and a temperature of 30 °C (independent of RH) for both *C. puteana* and *R. solani* therefore always resulted in limited fungal growth, while the optimal growing conditions were at 20 °C and 75% RH and at 25 °C and 80% RH for *R. solani* and at 20 °C and 75% RH for *C. puteana*. The method applied in this study offers an updated and broader alternative to classical and narrowly focused studies on fungal growth dynamics, and is well suited to efficiently assess the effect of environmental conditions on fungal growth.

**Electronic supplementary material:**

The online version of this article (10.1186/s43008-019-0009-3) contains supplementary material, which is available to authorized users.

## INTRODUCTION

Fungi are ubiquitous and grow even in the most extreme environments (Cooke 1968). Each fungal species grows under a particular range of environmental conditions. Most species achieve maximal growth only under specific environmental circumstances (Mislivec and Tuite 1970), referred to as the optimal growth conditions. Research on identifying these ranges is manifold and of use in various industries and research areas. Knowing the optimal growth conditions is, for instance, important when optimising industrial processes involving fungi, such as the production of cheese (Valík et al. 1999), antibiotics (Berdy 2005), paper (Torres et al. 2012) and biofuel (Vicente et al. 2009). Besides their applications, fungi also cause a remarkable amount of damage. Therefore, several studies have investigated the effects of environmental conditions on plant-pathogenic fungi, such as *Rhizoctonia solani* on potato and rice (Ritchie et al. 2009, Feng et al. 2017), *Puccinia purpurea* on sorghum (Dean et al. 2012) and *Helminthosporium fulvum* on tomato, rice, wheat, etc. (Ibrahim et al. 2011), and on fungi responsible for degradation of a wide range of building materials, such as gypsum drywall (Dedesko and Siegel 2015), wood (Erikson et al. 1990; Schmidt 2006) and other bio-based materials (Rijckaert et al. 1998; Jones and Brischke 2017). The environmental conditions also play an important role in hyphal exploration of material surfaces (leaves, wood, inert materials) and in the initial mycelial colonization of materials (Carlile et al. 2001; Li et al. 2014).

Studies investigating the impact of environmental conditions on fungal growth are mostly limited to a few conditions and/or fungal species, as a consequence of the expensive and time-consuming laboratory experiments. Some studies focus on the resistance of a certain substrate to fungal attack, assessing, for example, the resistance of crops (Pardo et al. 2005; Ibrahim et al. 2011) or of building materials, to fungi (Brischke and Thelandersson 2014). Fungal growth in these studies is usually measured as the mass loss of the studied substrate (Brischke and Rapp 2008; Osono 2015). Other studies examine the effects of the environmental conditions on fungal growth directly, by observing changes in the mycelium (Bonner and Fergus 1960; Boddy 1983; Pasanen et al. 1991). Most of these studies rely on simple experimental set-ups where all but one environmental condition is fixed and the fungus grows on an optimal growth medium. The techniques used for assessing fungal growth often involve microscopes and/or imaging devices to capture images or videos of growing fungi (Etheridge 1957; Ayerst 1969; Magan and Lacey 1984; Huang et al. 2001; Gock et al. 2003). Fungal growth characteristics, such as colony radius (Etheridge 1957; Boddy 1983; Pasanen et al. 1991), the growth rate (Bonner and Fergus 1960; Ayerst 1969; Magan and Lacey 1984; Gock et al. 2003) or the number of germinated spores (Tommerup 1983; Huang et al. 2001) can be derived directly from such images. Unfortunately, up to this day, these analyses are often performed manually, although an image analysis method is for instance available for measuring the mycelial area (Ancin-Murguzur et al. 2018). Such labour-intensive analyses hinder more detailed studies of fungal growth characteristics, such as the mean hyphal segment length and the total length of the mycelium, or the number of tips across the entire mycelium, even though the latter characteristics provide crucial insights into fungal growth. The financial and human resources needed to conduct detailed fungal growth analyses prevent the replication of experiments, such that the natural variability of fungal growth is often neglected. In addition, most relevant studies on detailed fungal growth dynamics often focus on small areas (Ramakrishna et al. 1993; Gougouli and Koutsoumanis 2013; van Laarhoven et al. 2015; Siripatrawan and Makino 2015) or the germination phase (Brunk et al. 2018), tracking growth of a few hyphae whose dynamics fail to represent the entire mycelium. Even though several studies compare the effect of environmental conditions on fungal growth over a certain period of time (Etheridge 1957; Magan and Lacey 1984; Pasanen et al. 1991; Nielsen et al. 2004), no elaborate time series analysis is performed to underpin the comparison.

Here we report on the influence of two of the most important environmental conditions, temperature and relative humidity (RH), on the growth dynamics of two fungal species using the (semi-) automated image analysis method of Vidal-Diez de Ulzurrun et al. (2015). *Coniophora puteana* and *Rhizoctonia solani*, were selected for assessment. Not only are these species economically important, they also have distinct growth dynamics (Vidal-Diez de Ulzurrun et al. 2015). Time series analysis is performed, based on the Granger Causality Test, the Mann Whitney Test and Dynamic Time Warping, to quantitatively compare the effect of environmental conditions, both within and between species. We aim to investigate how these environmental conditions influence the colony morphology of the two selected species and whether the fungal growth characteristics of the two fungal species respond differently to these different environmental conditions.

First, the two fungal species are presented and the image analysis method is explained, as well as the methods used for the time series analysis. Then, the overall behaviour of the measured fungal growth characteristics over time is presented, followed by a comparison between the growth dynamics of *C. puteana* and *R. solani* and an assessment of the effect of temperature and RH on the fungal growth dynamics of both species. This is followed by a discussion on the influence of different environmental conditions, the dynamics of several fungal growth characteristics and the added value of performing a time series analysis. We end with some conclusions and opportunities.

## METHODS

### Selected species

Two filamentous fungi were selected for this study: *Rhizoctonia solani* AG4-HG-I (Laboratory of Phytopathology of Ghent University, isolate from lettuce (Van Beneden et al. 2009): strain S010-1) and *Coniophora puteana* (strain MUCL 11662, BAM 15)*. R. solani* is a plant pathogenic fungus with a large range of hosts (Dean et al. 2012). It affects 5–10% of the total European sugar beet acreage (Büttner et al. 2003), causes up to 30% yield loss on affected potato crops (Tsror 2010), and up to 25% yield loss on affected cereals (MacNish and Neate 1996). The second species, *C. puteana,* is a common brown rot fungus responsible for the degradation of wood and building materials (Green III and Highley 1997, Schmidt 2006, Viitanen et al. 2010). It has been used for nearly 80 years as a test fungus for wood preservatives in Europe and is part of the current standards for testing wood preservatives and natural durability of wood species (CEN 1996, 2015).

### Experimental set-up

**Fig. 1:**
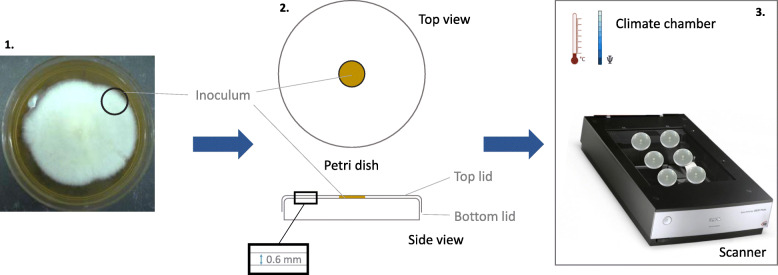
Schematic representation of the experimental set-up. A disc-shaped inoculum is taken from the periphery of the mother culture (1) and positioned at the centre of the bottom lid of a Petri dish (2). The top lid of the Petri dish is positioned on top of the bottom lid, as such restricting the height between top and bottom lids to 0.6 mm (2). Then, the Petri dishes are transferred to the flatbed scanner in a climate chamber (3)

Mother cultures were maintained on 8% malt agar substrate (2% agar Bacteriological No. 1; Oxoid, The Netherlands), 8% malt extract), for 3 days at 23 °C ± 2 °C and an RH of 65% ± 5% in a climate cabinet (CTS Pharma climatic test chamber Series CP; CTS Hechingen, Germany). The mother cultures were retained in Petri dishes of 9 cm diameter. After 3 days of growth, a disc-shaped inoculum of about 1 cm diameter was cut from the periphery of the mother culture and placed at the centre of the bottom lid of a Petri dish (Fig. 1). Finally, the top lid of the Petri dish was positioned on top of the bottom lid, as such restricting the height between top and bottom to 0.6 mm. In this way, vertical growth of the hyphae was limited and fungal growth was essentially restricted to two dimensions, which is a prerequisite for applying the image analysis procedure described below.

Images of the growing fungi were captured using a flatbed scanner (Epson perfection V750 Pro) on which two rows of three Petri dishes were mounted. The samples were positioned on the scanner immediately after inoculation as such allowing the capture of early growth. Growth was monitored for 62 h, as preliminary experiments showed that further expansion of the mycelium was limited after this time. During this period, the Petri dishes were scanned automatically every hour using VueScan (version 9.5.19). The images had a resolution of 1200 dpi (resulting in pixels of approximately 21 μm) and were automatically cropped to the growing area of the mycelium. The final images had dimensions of 2125 × 2125 pixels per sample, corresponding to approximately 4 × 4 cm^2^ and representing the central area of the Petri dish containing the initial inoculum.

The flatbed scanner was placed in a climate cabinet with controlled temperature and RH. The specific values for our experiments were selected in order to cover those used in earlier similar experiments (e.g. Bonner 1948; Bonner and Fergus 1960; Trinci 1969; Boddy 1983; Tommerup 1983; Magan and Lacey 1984; Pasanen et al. 1991; Ekesi et al. 1999; Bjurman and Wadsö 2000; Pardo et al. 2005; Brischke and Rapp 2008; Ritchie et al**.** 2009; de Oliveira et al. 2014), resulting in a full factorial experiment combining four temperatures (15, 20, 25 and 30 °C) and four relative humidity conditions (65, 70, 75 and 80% RH).

In summary, a total of 16 environmental conditions were tested. Six samples per environmental condition were initially prepared, as a maximum of six Petri dishes could be scanned simultaneously. Due to contamination in the form of dust and condensation, only four replicates per environmental condition could be included. For each environmental condition, the mean of the four replicates was calculated, and this for all growth characteristics.

### Quantification of fungal growth

The workflow for image analysis of fungal growth dynamics developed by Vidal-Diez de Ulzurrun et al. (2015) was adopted to assess the impact of environmental conditions on the growth dynamics of the selected species (Additional file 1: Figure S1). First, the inoculum was digitally removed from all images since growth on the agar disc cannot be captured (Fig. 2.2 b). Subsequently, a line detection algorithm (Lopez-Molina et al. 2015), implemented in MATLAB (Version R2016b; The Mathworks, Massachusetts USA), was applied to obtain binary ridge maps of the growing fungus (Fig. 2.2 c). These ridge maps were converted into graphs (Fig. 2.2 d) using Mathematica (Version 10.0; Wolfram Research, Illinois USA). The nodes in the graphs (Fig. 2.3) represent growing tips and junctions of hyphae, and the edges represent the hyphal segments connecting them. Finally, several morphologic characteristics were derived from these graphs, an overview of which is presented in Fig. 2.3. For a full explanation on the computation of these characteristics, we refer to Vidal-Diez de Ulzurrun et al. (2015). The number of tips was calculated as the number of nodes that are connected to a single other node. The mycelial area was calculated as the area of the smallest convex polygon in which all nodes fit. The mean growth and branching angles at a given time are calculated by taking the mean of all growth and branching angles present in the mycelium at that time. Note that the mean growth angle, the mean branching angle and the mean hyphal segment length are analysed only after 10 h of growth, because of their large standard deviation at the onset of growth, due to the presence of artificial nodes and edges along the inner boundary of the mycelium, which is intrinsic to the image analysis algorithm.

**Fig. 2:**
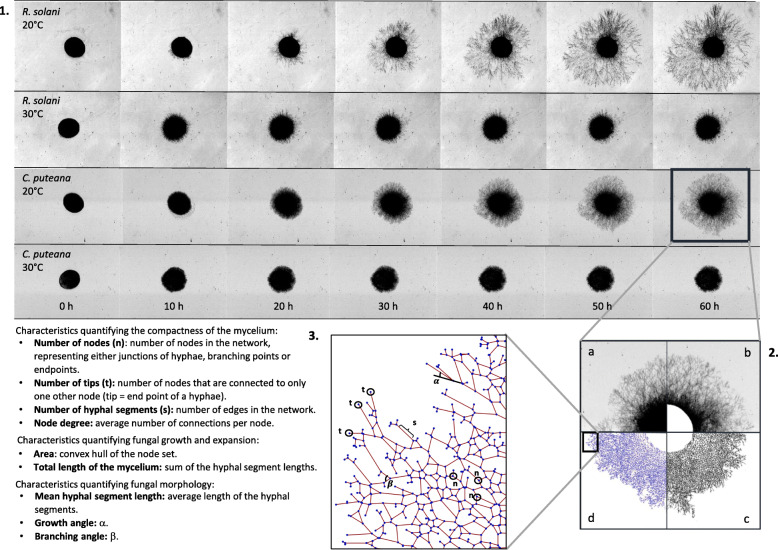
1. Images of *C. puteana* and *R. solani* in function of time at 75% RH in combination with 20 °C and 30 °C. 2. Summary of the entire process of characteristic extraction. a) Initial image; b) Pre-processed image; c) Binary ridge map; d) Graph. 3. Representation of characteristics quantifying fungal morphology

### Mathematical and statistical data analysis

The dataset consists of time series of the extracted fungal growth characteristics. For every environmental condition and characteristic, the mean value of four replicates was calculated at every time step. By mutually comparing the time series observed under different environmental conditions, we assessed the impact of temperature, RH and their combined effect on fungal growth. In order to identify discrepancies between time series, we used the Granger Causality Test (Granger 1969), the Mann Whitney Test (Mann and Whitney 1947), and we computed the Dynamic Time Warping distance (Berndt and Clifford 1994).

The Granger Causality Test (GCT, Granger 1969) is based on the idea that if the prediction of one time series is improved by incorporating the information of a second time series, the latter is said to have a causal influence on the former (Guo et al. 2010). When this is not the case, one can assume that the time series show a significantly different behaviour. The Mann Whitney test (MWT) assesses whether the medians of two distributions differ significantly (Mann and Whitney 1947). In this paper, the MWT was used to verify whether the difference of the medians of two time series is equal to zero. Dynamic Time Warping (DTW) is a well-known method for finding an optimal alignment between two given time series. In this paper, DTW was used to indicate how distinct the growth curves corresponding to different conditions are, compared to the average DTW distance found between replicates of those conditions. Additionally, autocorrelation tests were performed on the four replicates for every characteristic to see whether two time series indeed behave differently, or whether the abovementioned tests only pinpointed them as being different due to random fluctuations in the data. All analyses were performed in Mathematica (Version 10.0, Wolfram Research, Illinois USA).

## RESULTS

### General fungal growth dynamics

**Fig. 3:**
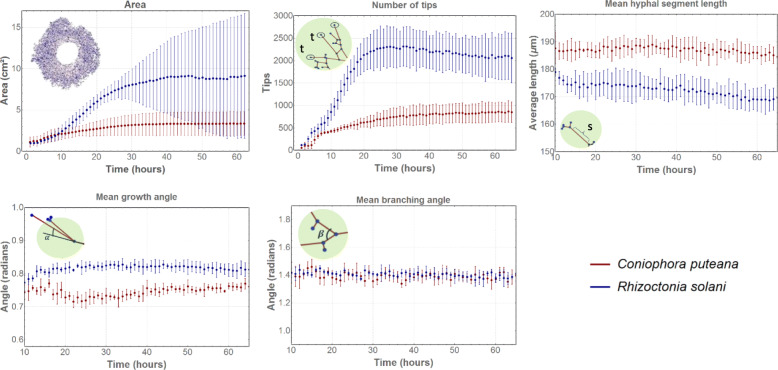
Fungal growth characteristics in function of time at 25 °C and 80% RH for *C. puteana* (red) and *R. solani* (blue). The dots represent the average value of four replicates, with the bars indicating the standard deviation

Several fungal growth characteristics were assessed over time for each species (Fig. 2.3)*.* An example of the main fungal growth characteristics in function of time is given in Fig. 3, showing the results obtained at 25 °C and 80% RH. Under this condition, the number of tips increased over the first 20 h for *Coniophora puteana* and *Rhizoctonia solani*, after which growth ceased. The mycelial area, on the other hand, continued to increase gradually even beyond those first 20 h. Across all conditions, a few general trends could be inferred: the mycelial area and the number of tips increased over time in a sigmoidal manner, whereas the mean hyphal segment length remained fairly constant. The growth and mean branching angles fluctuated irregularly over time.

Since the mean hyphal segment length remains almost constant, the mean value was calculated for each condition (Table 1), and averaged over all conditions we obtained 176.5 ± 7.8 μm (*R. solani)*. There are no differences in mean hyphal segment length across the 16 conditions, within the accuracy determined by the scanner resolution (approx. 21 μm). The overall mean hyphal segment length for *R. solani* is thus 176.5 ± 21 μm. A similar approach was followed for the mean hyphal segment length for *C. puteana,* calculated as 183.2 ± 21 μm, which is not significant given the limits of scanner resolution.

**Table 1:** Mean hyphal segment length of *R. solani* averaged over four replicates and over time

RH/temp	65% RH	70% RH	75% RH	80% RH
*15 °C*	181.1 ± 4.4	174.6 ± 3.9	181.7 ± 2.9	180.0 ± 4.7
*20 °C*	181.5 ± 7.4	181.9 ± 9.8	168.1 ± 5.5	178.1 ± 3.5
*25 °C*	178.7 ± 10.7	180.0 ± 11.3	178.0 ± 3.3	172.0 ± 4.5
*30 °C*	170.6 ± 3.2	172.2 ± 9.2	168.6 ± 2.5	176.3 ± 2.6

**Fig. 4:**
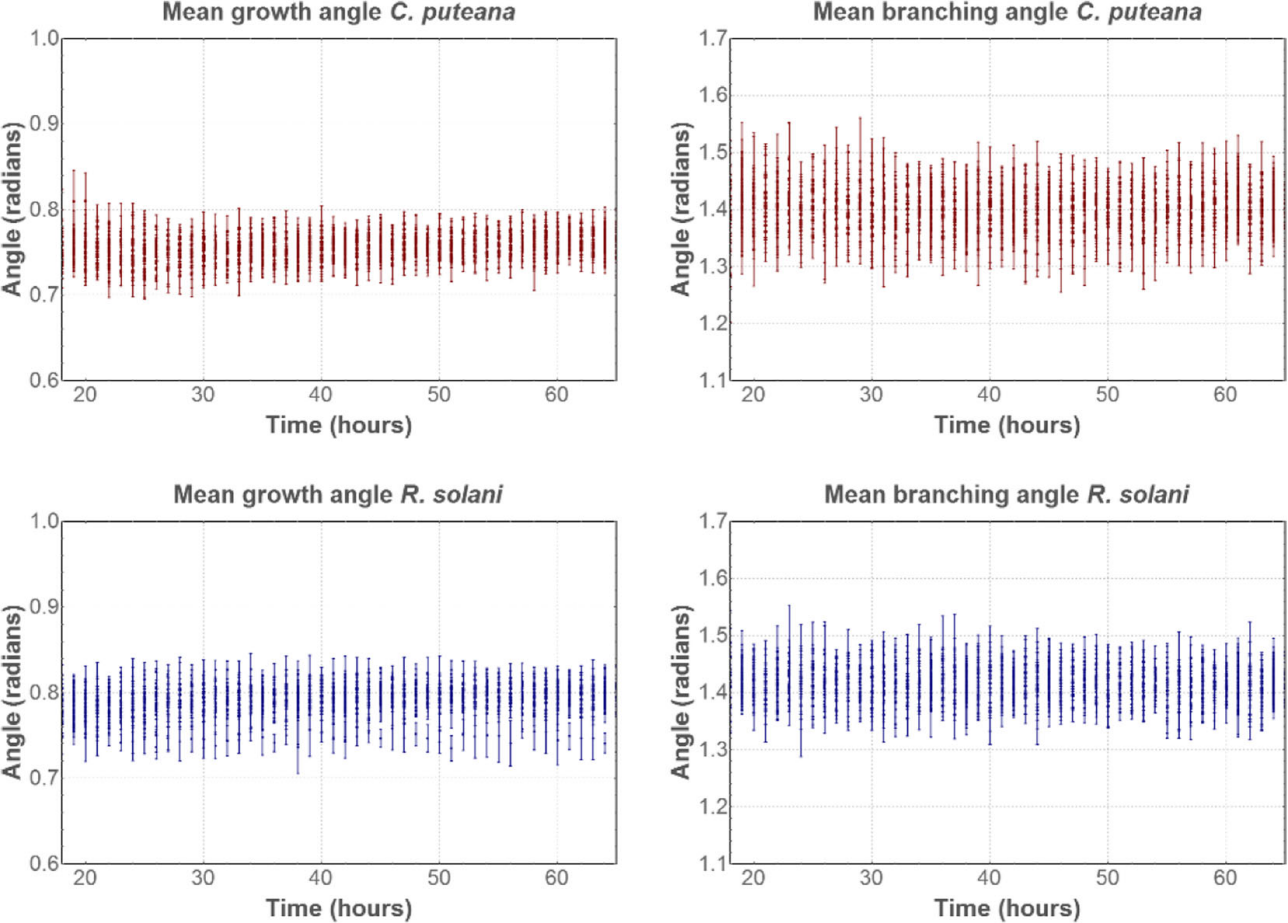
Mean growth and branching angles of *C. puteana* (red) and *R. solani* (blue), for four replicates of 16 conditions. The mean growth and branching angles at a given time were calculated by taking the mean of all growth and branching angles present in the mycelium at that time

The mean growth angle and mean branching angle seem to vary randomly in function of time, yet within rather narrow boundaries (Fig. 4). Mean branching angles varied between 1.22–1.56 rad (*C. puteana)* and 1.28–1.58 rad (*R. solani*), whereas mean growth angles varied between 0.69–0.82 rad (*C. puteana)* and 0.70–0.86 rad (*R. solani*).

### Comparison of growth dynamics

Table 2 compares the fungal growth characteristics of the two species, for the 16 environmental conditions. Based on the MWT, the growth characteristics of both fungi were found to differ for nearly every characteristic and condition. For example, at 25 °C and 80% RH (Fig. 3), one can clearly see a difference in absolute value between the species for several characteristics: the mycelial area and the number of tips formed by *R. solani* were more than twice as large than those of *C. puteana*. Based on the results of the GCT, the differences between *C. puteana* and *R. solani* were more pronounced for some characteristics than for others. The mycelial area, the number of tips, the mean edge length, and the mean growth and branching angles differed between the two species in at least 10 of the 16 tested conditions (Table 2). The autocorrelation for these characteristics indicated clear trends, with the exception of the mean growth and branching angles whose autocorrelation was only 0.2. This demonstrates that these two characteristics fluctuated randomly (Fig. 3), making the results of the GCT for the growth and mean branching angles irrelevant. Nonetheless, a difference in mean growth angle between the two species can be assessed visually in Fig. 3.

**Table 2:** Comparison of growth characteristics of C. puteana and *R. solani* for 16 combinations of temperature and RH

Environmental condition	Area		Number of tips		Mean hyphal segment length (after 10 h)		Mean growth angle (after 10 h)		Mean branching angle (after 10 h)	
	GCT	MWT	GCT	MWT	GWT	MWT	GWT	MWT	GWT	MWT
15 °C, 65% RH	x	x	x	x	x	x	x	x	x	x
15 °C, 70% RH			x	x	x	x		x	x	x
15 °C, 75% RH	x	x		x	x	x	x	x	x	x
15 °C, 80% RH	x	x	x	x	x	x		x	x	x
20 °C, 65% RH	x	x	x	x	x	x		x	x	x
20 °C, 70% RH	x	x	x	x	x	x	x	x	x	x
20 °C, 75% RH	x	x	x	x	x	x		x	x	x
20 °C, 80% RH		x		x	x	x	x	x	x	x
25 °C, 65% RH	x	x	x	x	x	x	x	x	x	x
25 °C, 70% RH	x	x	x	x	x	x	x	x	x	x
25 °C, 75% RH	x	x	x	x		x	x	x	x	x
25 °C, 80% RH	**x**	**x**	**x**	**x**	**x**	**x**	**x**	**x**	**x**	**x**
30 °C, 65% RH		x	x	x	x	x	x	x	x	x
30 °C, 70% RH		x		x	x	x	x	x	x	x
30 °C, 75% RH		x		x	x	x	x	x	x	x
30 °C, 80% RH		x	x	x	x	x		x	x	x

x: *p*-value of the GCT is > 0.05, meaning that the time series for C. puteana cannot be used to predict the corresponding series for R. solani and/or vice versa (= time series are different). x: *p*-value < 0.05 for the MWT, meaning that the median difference between two corresponding time series is not equal to zero (= time series are different). The GCT and the MWT for the environmental condition at 25 °C and 80% RH are highlighted for the sake of comparison with Fig. 3*.*

### Effect of temperature and relative humidity on fungal growth

**Fig. 5:**
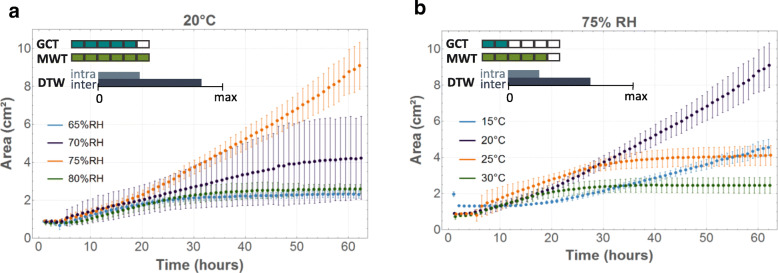
Evolution of mycelial area (cm^*2*^) over time for *C. puteana* at 20 °C (**a**) and 75%RH (**b**), showing a clear combined effect of temperature and RH. The dots represent the mean values over four replicates, with the bars indicating the standard deviation. For each graph, six pairwise GCT and MWT were performed between the four conditions represented in that graph. The two colour scales indicate for how many of those pairwise comparisons a difference was found according to GCT (blue) and MWT (green). The third scale represents the difference between the average DTW distance between the four conditions represented in the graph and a reference ground value, being the average DTW distance over the replicates in that graph. The bigger the bar, the more difference there is between conditions. When the DTW scale is negligibly small, the difference between conditions is insignificant compared to the reference DTW difference between replicates

Temperature and RH often had a combined influence on fungal growth dynamics. At 20 °C and at 25 °C (*C. puteana)* and at 15 °C and 25 °C (*R. solani),* the growth curves representing the evolution of the mycelial area over time clearly differed across the four RHs. For instance, for *C. puteana* at 20 °C and 75% RH, the mycelial area was more than twice as large than at an RH of 70%, while only limited growth occurred at 65 and 80% RH (Fig. 5 a). Similarly, at a RH of 75%, growth curves differed across the four temperatures, which was confirmed by DTW and most of the GCT and MWT (Fig. 5b).

**Fig. 6:**
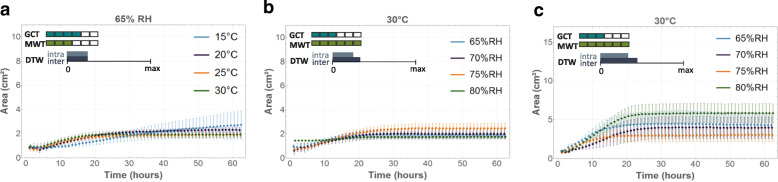
Evolution of mycelial area (cm^*2*^) over time for *C. puteana* at 65% RH (**a**) and 30 °C (**b**) and *R. solani* at 30 °C (**c**), showing limited growth. The dots represent the mean values over four replicates, with the bars indicating the standard deviation. For each graph, six pairwise GCT and MWT were performed between the four conditions represented in that graph. The two colour scales indicate for how many of those pairwise comparisons a difference was found according to GCT (blue) and MWT (green). The third scale represents the difference between the average DTW distance between the four conditions represented in the graph and a reference ground value, being the average DTW distance over the replicates in that graph. The bigger the bar, the more difference there is between conditions. When the DTW scale is negligibly small, the difference between conditions is insignificant compared to the reference DTW difference between replicates

In contrast, an RH of 65% (independent of temperature) for *C. puteana* and a temperature of 30 °C (independent of RH) for both *C. puteana* and *R. solani* always resulted in limited fungal growth, as confirmed by DTW and the GCT (Fig. 6).

**Fig. 7:**
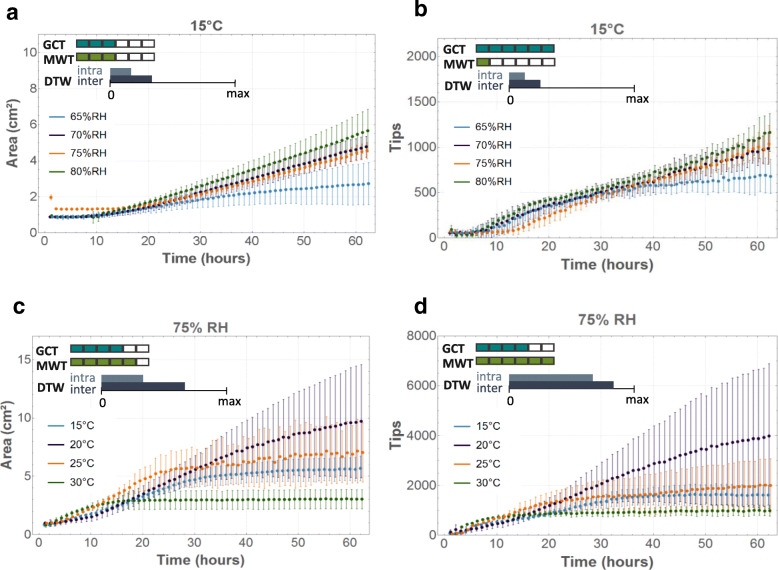
Evolution of mycelial area (cm^*2*^) and the number of tips over time for *C. puteana* (**a**, **b**) and *R. solani* (**c**, **d**). The dots represent the mean values over four replicates, with the bars indicating the standard deviation. For each graph, six pairwise GCT and MWT were performed between the four conditions represented in that graph. The two colour scales indicate for how many of those pairwise comparisons a difference was found according to GCT (blue) and MWT (green). The third scale represents the difference between the average DTW distance between the four conditions represented in the graph and a reference ground value, being the average DTW distance over the replicates in that graph. The bigger the bar, the more difference there is between conditions. When the DTW scale is negligibly small, the difference between conditions is insignificant compared to the reference DTW difference between replicates

The influence of temperature and RH on the evolution of the number of tips was similar as for the mycelial area (Fig. 7). Some environmental conditions caused substantially more variation, as was the case for the mycelial area and the number of tips at 20 °C and 70% RH and 25 °C and 75% RH for both fungi (Fig. 7c, d), and for the mycelial area at 25 °C and 80% RH. The optimal growth conditions for mycelium development over an inert surface, defined as the conditions where the largest area and highest number of tips were reached after 62 h, occurred at 20 °C and 75% RH and at 25 °C and 80% RH for *R. solani* AG4-HG-I and at 20 °C and 75% RH for *C. puteana* MUCL 11662 (Additional file 2: Figure S2, Additional file 3: Figure S3, Additional file 4: Figure S4 and Additional file 5: Figure S5).

## TAXONOMY

### Material examined

*Coniophora puteana* strain MUCL 11662, BAM 15, Belgium, Louvain-la-Neuve, preserved at Mycothèque de l’Université catholique de Louvain, culture collection acronym and accession number: MUCL 11662.

*Rhizoctonia solani* AG4-HG-I strain S010-1, Belgium, Ghent 9000, isolate from lettuce (Van Beneden et al. 2009), preserved at the Laboratory of Phytopathology of Ghent University, culture collection acronym and accession number: S010-1.

## DISCUSSION

Temperature and RH are known to significantly affect fungal growth dynamics (Bonner 1948; Bonner and Fergus 1960; Mislivec and Tuite 1970; Pasanen et al. 1991). This has been observed both in laboratory conditions (Boddy 1983; Gougouli and Koutsoumanis 2013; Tommerup 1983; van Laarhoven et al. 2015; Huang et al. 2001; Gock et al. 2003) and in natural environments (Kauserud et al. 2010; Gange et al. 2011). However, such studies rarely assess the combined effect of temperature and RH.

A study by Ciliberti et al. (2015), considering the influence of temperature and RH simultaneously on grape rot (*Botrytis cinerea*), points at an interaction effect between both factors. A combined effect of temperature and RH on radial growth (Bonner and Fergus 1960; Pasanen et al. 1991) and germination time (Bonner 1948; Mislivec and Tuite 1970) can be noted from an examination of the results presented in these studies, although the authors do not mention this as such. Our study confirms that there is indeed a combined effect of temperature and RH on several fungal growth characteristics, for both *C. puteana* and *R. solani*. Furthermore, the optimal temperature ranges for a given species often differ between different studies. For instance, for *C. puteana* 22–25 °C (Schmidt et al**.** 2002), 20 °C (Etheridge 1957), 20–25 °C (Seehann and Riebesell 1988), 28 °C (Wälchli 1977) and 20–32 °C (Jones and Brischke 2017) were reported as optimal temperature ranges. This wide variety can partly be explained by neglecting the effect of RH, as Etheridge (1957) and Seehann and Riebesell (1988) do not mention the RH used, while Schmidt et al. (2002) maintain a non-specified high RH. Also, the genetic variation between the strains could have an influence on the optimal temperature range. Schmidt et al**.** (2002) reported an optimal temperature of 22.5 °C for 7 of the 15 isolates of *C. puteana*, while a temperature of 25 °C was optimal for 8 isolates*.* For *R. solani*, a wide range of optimal temperatures is reported as well, including 20 and 25 °C (Anguiz and Martin 1989), 20–25 °C (Ritchie et al. 2009), 22–25 °C (Chand and Logan 1983), 25 °C (Grosch and Kofoet 2003), 24–27 °C (de Oliveira et al. 2014), 25–30 °C (Harikrishnan and Yang 2004) and 30–35 °C (Baird et al. 1996). We found that the optimal conditions for mycelial development over an inert surface for *C. puteana* were reached at 20 °C and 75% RH and for *R. solani* at 20 °C and 75% RH, and 25 °C and 80% RH.

Fungi can grow at very low levels of RH if water is available on the surface (Pasanen et al. 1991). When only a limited amount of water is available, *in casu* in our set-up, RH plays a significant role in mycelial development. In this paper, an RH of 65% was limiting for mycelium development over the inert Petri dish surface, irrespective of the temperature, for *C. puteana.* None of the tested RH conditions was limiting for *R. solani.* This does not preclude that RH is never the main limiting factor for *R. solani*, but only that the ranges for which we tested might not have included an RH low enough to limit the mycelium development of *R. solani.* A temperature of 30 °C was limiting for fungal growth, irrespective of the RH, both for *C. puteana* and *R. solani.* This can possibly be explained by a drying out of the exploring hyphae at this temperature. When water availability is not an issue, optimal temperatures up to 32 °C for *C. puteana* and 35 °C for *R. solani* have been reported (Baird et al. 1996; Jones and Brischke 2017).

Growth of filamentous fungi over time is typically sigmoidal, with a lag phase at lower growth rates, followed by an exponential growth phase, and a brief stationary phase, after which the fungus dies (Trinci 1969; Trinci 1974; Montini et al. 2006; Meletiadis et al. 2001). The growth curves presented in this paper (Additional file 2: Figure S2- Additional file 5: Figure S5) are sigmoidally shaped when temperature and/or RH do not limit growth, and correspond to those in Montini et al. (2006) and Meletiadis et al. (2001). The set-up we used does not allow distinguishing between living and dead hyphae. When the mycelial area or the number of tips do no longer increase, growth has ceased. Fungal growth ceased either early on in the experiment, indicating that the environmental conditions had a limiting effect on fungal growth (for instance for *R. solani* at 30 °C, as shown in Fig. 6), or only after a large mycelial area had formed, indicating that the substrate was the limiting factor (for instance for *R. solani* at 25 °C and 80% RH, as shown in Additional file 3: Figure S3).

Very few studies on fungal growth dynamics examine the mean hyphal segment length of a fungal network, as most determine the hyphal growth unit, which is the total length of the mycelium divided by the total number of tips (Carlile et al. 2001). The values for mean hyphal segment length determined in this paper, being 183.2 ± 21.0 μm (*C. puteana*) and 176.5 ± 21.0 μm (*R. solani*), do agree with those presented by Vidal-Diez de Ulzurrun et al. (2015). *Coniophora puteana* and *R. solani* have similar mean hyphal segment lengths, which are comparable to those of *Phanerochaete velutina* (186.3 ± 7.4 μm) and *Penicillium lilacinum* (168.9 ± 23.7 μm) (Vidal-Diez de Ulzurrun et al. 2015). In contrast, the mean hyphal segment lengths of *Trichoderma viride* (209.1 ± 12.2 μm) and *Mucor hiemalis* (117.0 ± 84.0 μm) are significantly longer and shorter, respectively (Hutchinson et al. 1980; Vidal-Diez de Ulzurrun et al. 2015). It seems that the mean hyphal segment length (i.e. the mean length of the hyphal segments) can be a distinguishing morphological characteristic between fungal species.

**Fig. 8:**
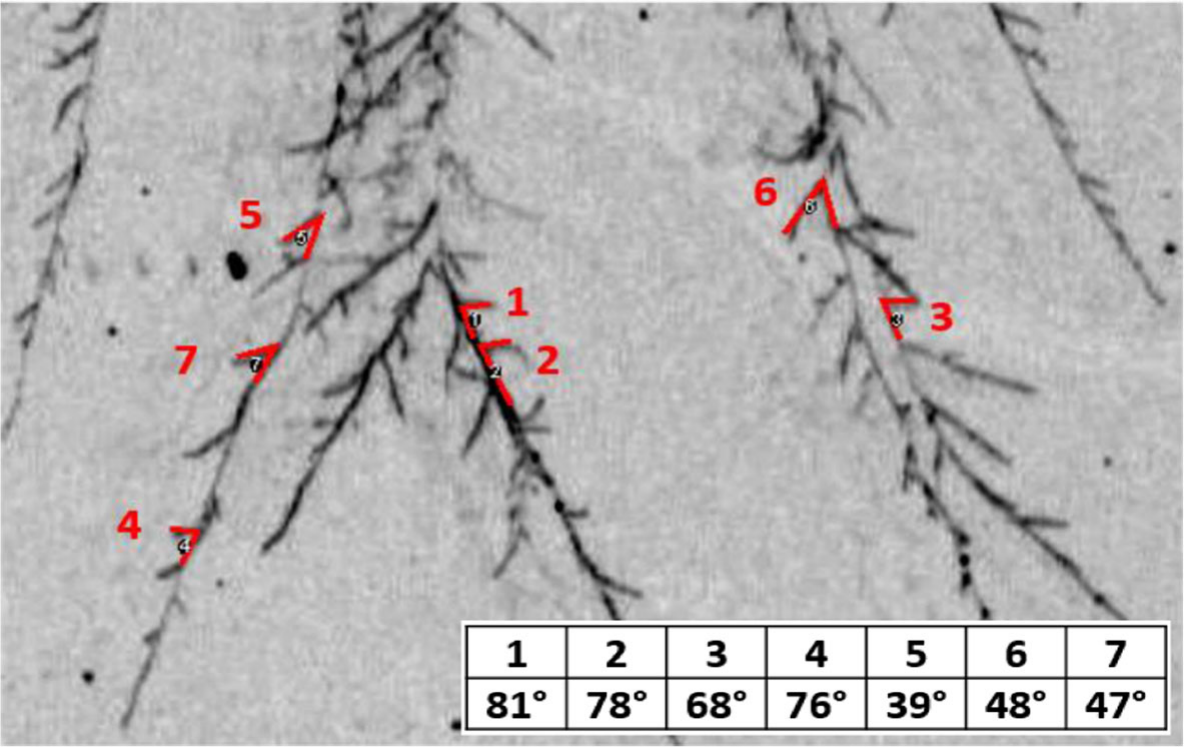
Manual indication of branching angles on *R. solani* hyphae at 20 °C and 75% RH. Branching angles are highlighted and calculated with ImageJ (Schindelin et al. 2015)

Generally, filamentous fungi form branches at approximately right angles to the parent hyphae (Carlile et al. 2001). For *R. solani* specifically, right-angled branching angles were noted in young mycelium by Kamel et al. (2009). Here, mean branching angles were found between 1.22–1.56 rad (*C. puteana)* and 1.28–1.58 rad (*R. solani*), which are overall notably smaller than right angles (1.57 rad). This can also be observed in Fig. 8, where some branching angles of *R. solani* are highlighted and calculated with ImageJ (Version 2, Fiji).

**Fig. 9:**
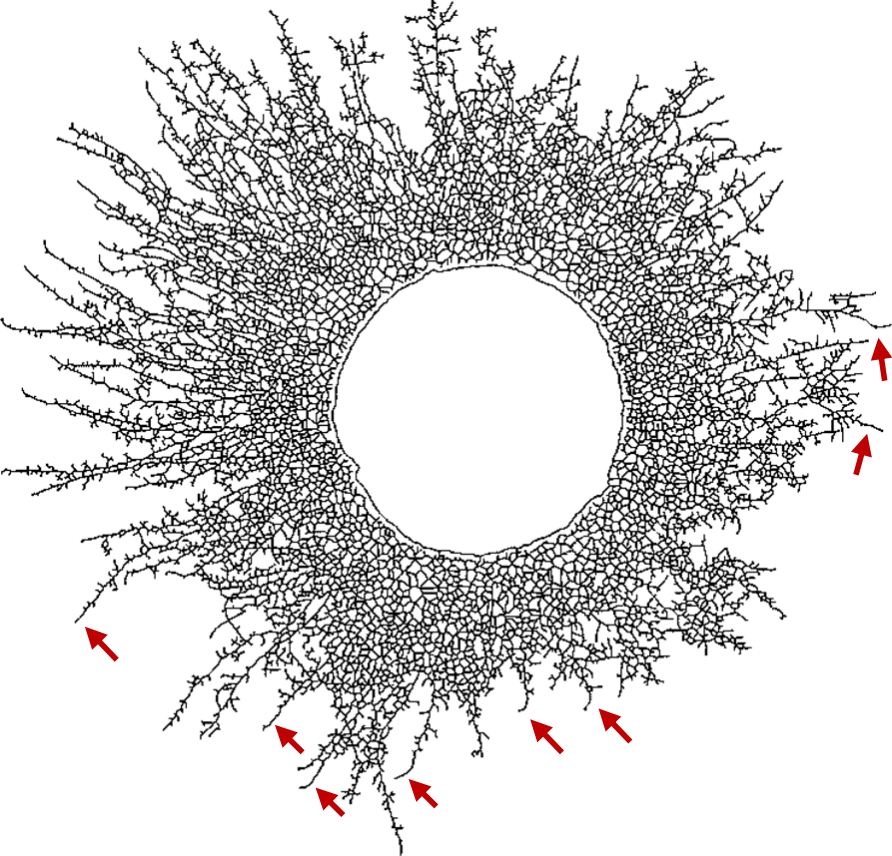
Elongation of hyphal segments at the periphery of the mycelium. Binary image, representing the mycelium of *R. solani* after 30 h of growth, at 25 °C and 80% RH

Hyphae have the tendency to maintain a certain direction of growth (Riquelme et al. 1998). The growth angle is defined as the difference between the angle of a segment and the angle of its preceding segment (Fig. 2), and as such gives an indication of the growth direction of the hyphae. In general, hyphae will maintain their direction while elongating and small growth angles will be found. A low value for the mean growth angle therefore indicates that there are almost no changes in the growth direction, while larger values possibly imply a complex network full of branches. The mean growth angles in this paper were similar for *C. puteana* (0.70–0.86 rad) and *R. solani* (0.69–0.82 rad), and indicate that branching happens frequently (Fig. 4). Vidal-Diez de Ulzurrun et al. (2015) report similar values for the mean branching angle and the mean growth angle for *C. puteana* and *R. solani*. When investigating the periphery of the mycelium, clear hyphal elongation can be noted. For example, for *R. solani* at 25 °C and 80% RH, the number of edges, tips and nodes did no longer increase after 20 h, while the area continued to increase (Fig. 3). Given that the mycelial area was measured as the convex hull of the mycelium and no new tips were formed, this indicates that only the length of the hyphal segments increased. Indeed, at the end of the growing period, hyphal elongation occurred at the periphery of the mycelium while branching subsided (Fig. 9). This elongation of the so-called “leading hyphae” (hyphae extending at the mycelium edge (Vinck et al. 2005, Lew 2011)) is typical for nutrient-poor conditions, where branching is suppressed to maximise the extension of these leading hyphae (Esser and Lemke 1995).

Under optimal growth conditions, growth characteristics differed significantly between replicates, see, for example, the large standard deviation for the mycelial area at 20 °C and 70% RH, 25 °C and 80% RH (both fungi) and at 25 °C and 75% RH (*R. solani)* (Additional file 2: Figure S2-Additional file 3: Figure S3), and for the number of tips at 20 °C and 70% RH (both fungi), 25 °C and 80% RH (*C. puteana*) and 25 °C and 75% RH (*R. solani)* (Additional file 4: Figure S4-Additional file 5: Figure S5). Even though the set-up guaranteed as little variation between the samples as possible, small differences might have occurred in, for example, the amount of substrate, the initial humidity of the agar disc and the number of hyphae on the agar disc, while natural variability obviously always has to be taken into account.

### Temporal changes

Various studies assessing the effect of environmental conditions on fungal growth characteristics neglect time. They either assess fungal growth characteristics after a fixed number of days (Bonner and Fergus 1960; Ibrahim et al. 2011; Ciliberti et al. 2015; Pardo et al. 2005) or use average growth rate (Ayerst 1969; Boddy 1983; Tommerup 1983; van Laarhoven et al. 2015), yet taking temporal changes into account is important. For instance, for an RH of 65% the mycelial area of *R. solani* after 62 h was the same at 15 °C and 20 °C, but at 20 °C it reached this value earlier (Additional file 3: Figure S3). Therefore, computing this characteristic after 60 h only would not allow for the detection of the different behaviour. In most studies the growth curves are usually visually compared (Pasanen et al. 1991; Ramakrishna et al. 1993; Nielsen et al**.** 2004). Clearly, a time series analysis, as applied in this study allows for an objective comparison of fungal growth behaviour as a function of time. This enables a more thorough and objective comparison of the influence of environmental conditions, and allows for verifying whether or not there is a combined effect of temperature and RH on fungal growth dynamics.

## CONCLUSIONS

The method presented here enables an objective and in-depth analysis of the effect of environmental conditions on various fungal growth characteristics, to be carried out in a rather short period of time (62 h). It offers an updated and broader alternative to the classical studies on fungal growth dynamics with narrow observation areas and/or a limited number of characteristics. Moreover, it can be performed with low-cost imaging devices, such as scanners. Comparing fungal growth based on time series analysis is an innovative approach which enabled a more thorough and objective comparison of the influence of environmental conditions, and allowed to verify the combined effect of temperature and RH on fungal growth dynamics. RH plays an important role in mycelial development when a limited amount of water is available. An RH of 65% (*C. puteana*) and a temperature of 30 °C (*C. puteana* and *R. solani)* resulted in limited fungal growth. Optimal conditions for mycelial development over an inert surface occurred at 20 °C and 70% RH (*C. puteana*) and at 20 °C and 75% RH, and 25 °C and 80% RH (*R. solani*). Several fungal growth characteristics showed sigmoidal growth over time, which is typical for filamentous fungi, while the mean hyphal segment length remained constant over time. By measuring several fungal growth characteristics, elongation of the “leading hyphae” could also be observed.

The method presented here allows for a quantitative and thus objective mutual comparison of different growth characteristics as a function of time and could be deployed for a number of research topics involving fungal growth dynamics, such as testing of different growth substrates or for finding differences in phenotype between genetic variants of the same species.

## Additional files

Additional file 1: **Figure S1.1.** and **Figure S1.2.** Workflow for image analysis of fungal growth dynamics developed by Vidal-Diez de Ulzurrun et al. (2015). (PDF 524 kb)

Additional file 2: **Figure S2.** Evolution of mycelial area (cm^2^) over time for *C. puteana*. (PDF 445 kb)

Additional file 3: **Figure S3.** Evolution of mycelial area (cm^2^) over time for *R. solani*. (PDF 604 kb)

Additional file 4: **Figure S4.** Evolution of the number of tips over time for *C. putean*a. (PDF 459 kb)

Additional file 5: **Figure S5.** Evolution of the number of tips over time for *R. solani*. (PDF 594 kb)

## Abbreviations

DTW: Dynamic Time Warping
GCT: Granger Causality Test
MWT: Mann Whitney Test
RH: Relative humidity
