# Supplementary material for: Analysis of spatio-temporal fungal growth dynamics under different environmental conditions
Source: IMA Fungus. 2019 Jun 21;10:7. doi: 10.1186/s43008-019-0009-3 (PMC7325663; doi:10.1186/s43008-019-0009-3)
Supplement: Supplementary file 1 — Figure S1.1. and Figure S1.2. Workflow for image analysis of fungal growth dynamics developed by Vidal-Diez de Ulzurrun et al. (2015). (PDF 524 kb) [file 43008_2019_9_MOESM1_ESM.pdf]

4-step process to extract fungal growth measures from the initial images (Fig. S1.2 a).

Step 1: Removing noise in the images, such as droplets of agar and the initial inoculum (Fig. S1.2 b)

Step 2: Extracting the fungal network.
A line detection algorithm (Lopez-Molina *et al*., 2015) is used to extract a thin binary ridge map from each image, which represents the fungal network (Fig. S1.2 c). Binary ridge maps for *R. solani* growing *in vitro* are represented in Fig. S1.1.

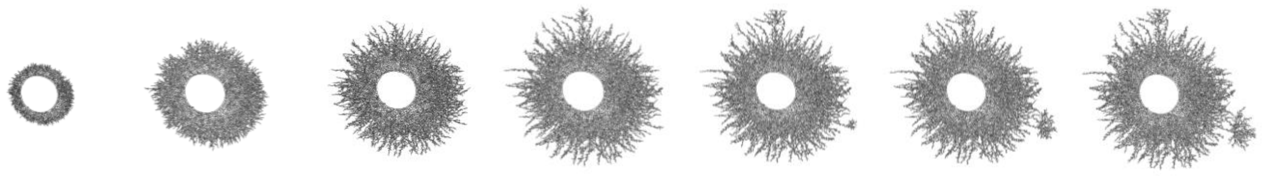

Fig. S1.1 Evolution of the fungal network of *R. solani,* extracted using a line detection algorithm (Lopez-Molina et al., 2015). The pictures represent the growth from 10 to 72 hours at intervals of 10 hours.

Step 3: Converting the ridge map into a graph. The MorphologicalGraph function of Mathematica (Version 10.0, Wolfram Research Inc., USA) converts images into mathematical graphs (Fig. S1.2 d): nodes represent junctions (intersections) of hyphae and tips (apices) of the mycelium and the edges represent the hyphal segments connecting them.

Step 4: Extracting fungal measures. Using the information contained in the graphs, we can compute some of the most important fungal growth characteristics: the total number of tips, the area of the mycelium, etc.

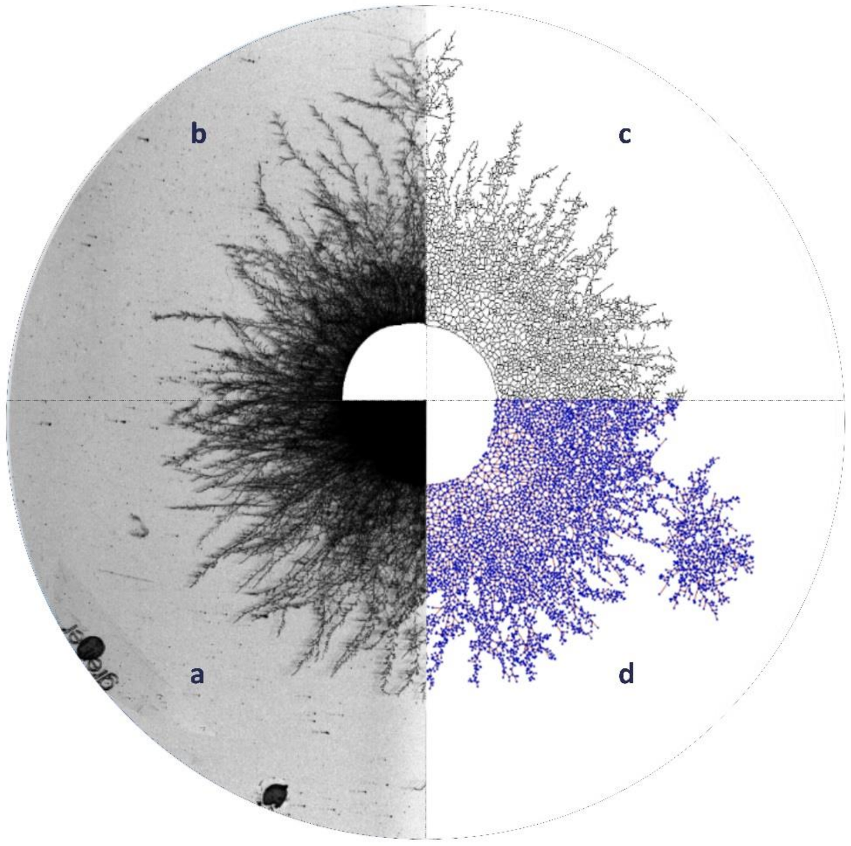

Fig. S1.2 Visual representation of the entire process of fungal growth measure extraction from an image of *R. solani*. a) Initial image; b) Preprocessed image; c) Binary ridge map; d) Mathematical graph.

Fig. S1 Workflow for image analysis of fungal growth dynamics developed by Vidal-Diez de Ulzurrun et al. (2015)
