# Supplementary figures and images for: Analysis of spatio-temporal fungal growth dynamics under different environmental conditions
Source: IMA Fungus. 2019 Jun 21;10:7. doi: 10.1186/s43008-019-0009-3 (PMC7325663; doi:10.1186/s43008-019-0009-3)

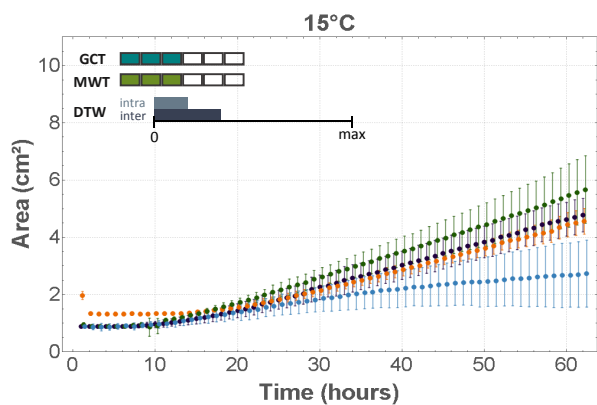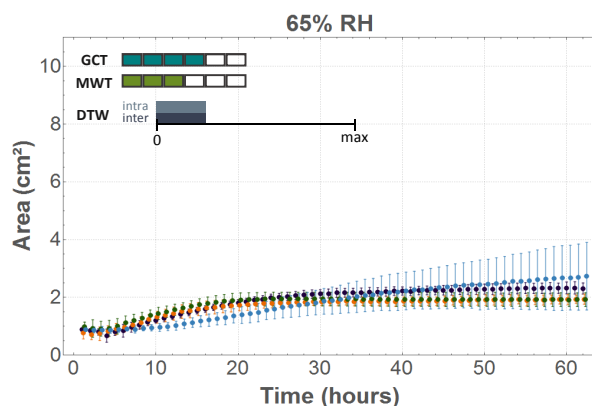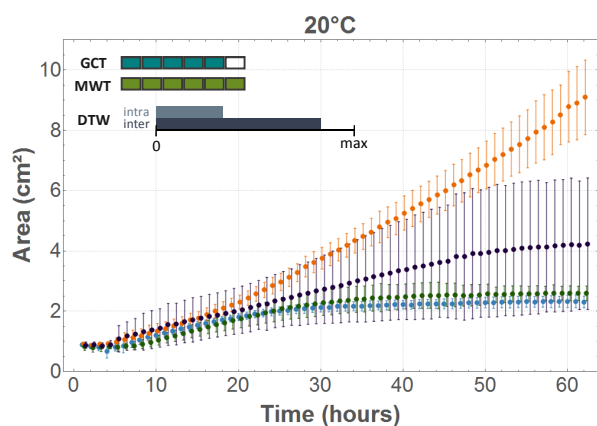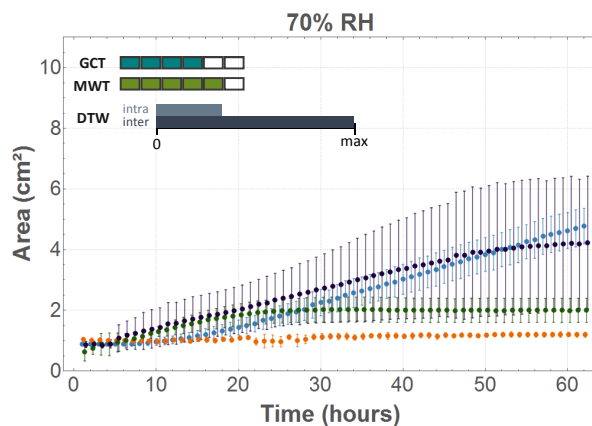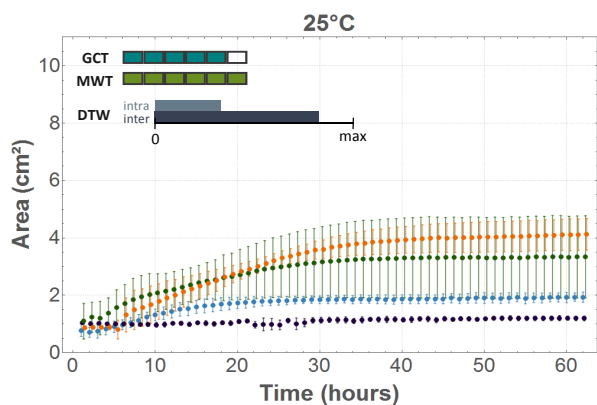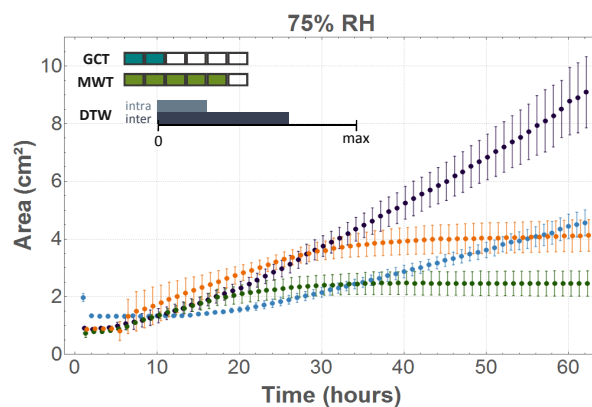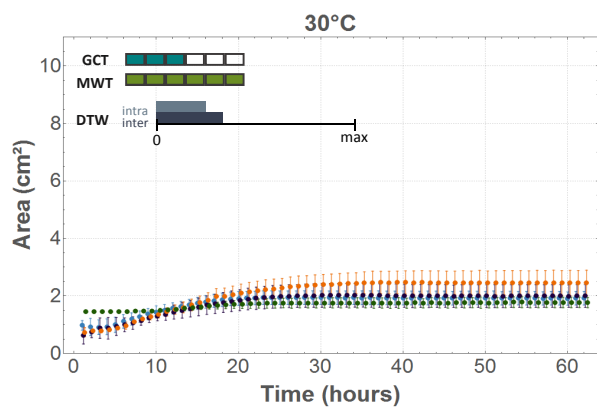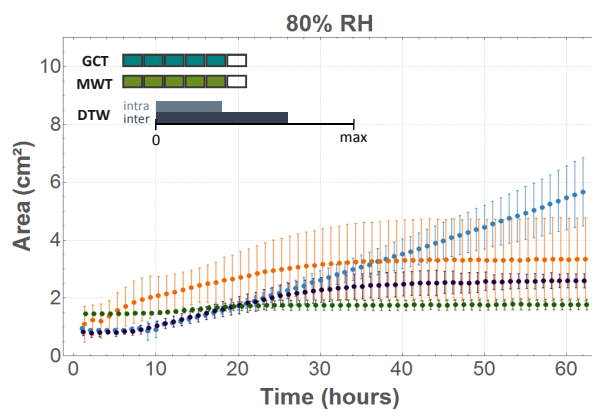

— 65%RH  
— 70%RH  
— 75%RH  
— 80%RH

— 15°C  
— 20°C  
— 25°C  
— 30°C

Supplement: Supplementary file 2 — Figure S2. Evolution of mycelial area (cm2) over time for C. puteana. (PDF 445 kb) [file 43008_2019_9_MOESM2_ESM.pdf]

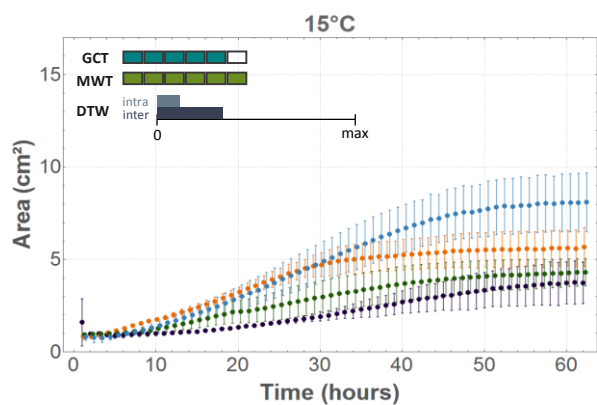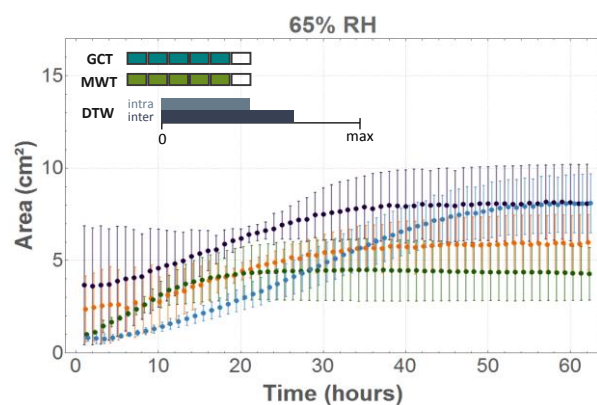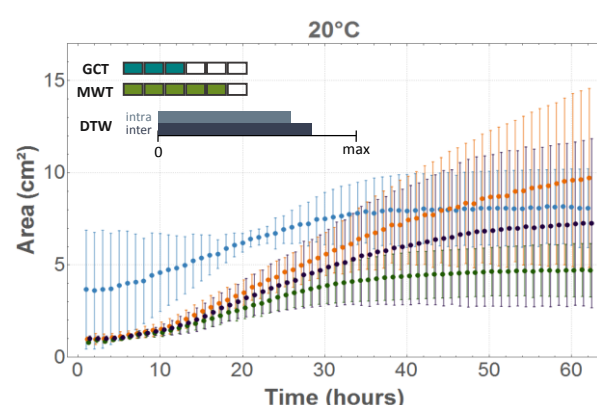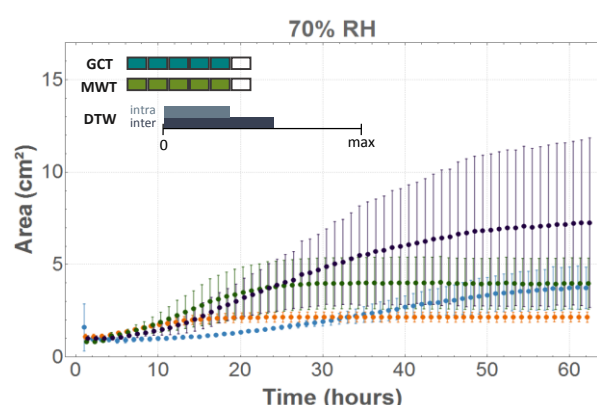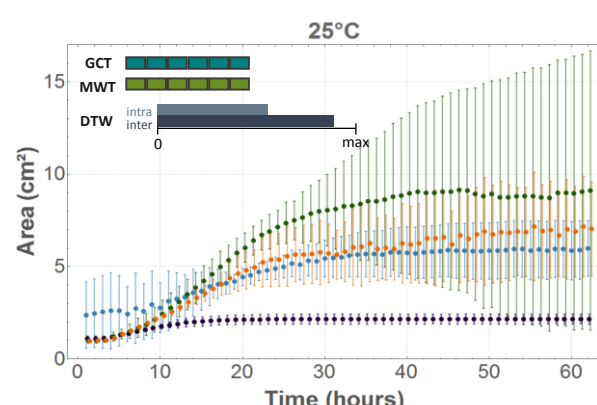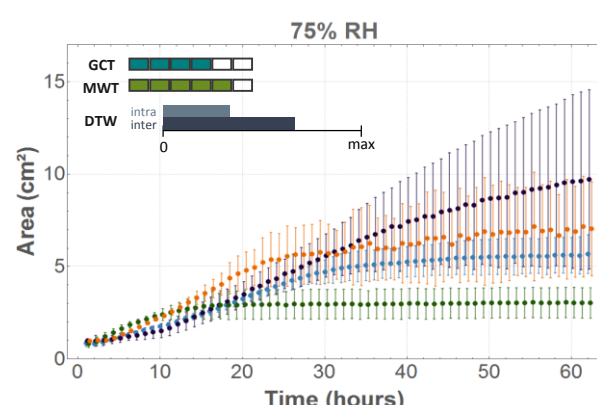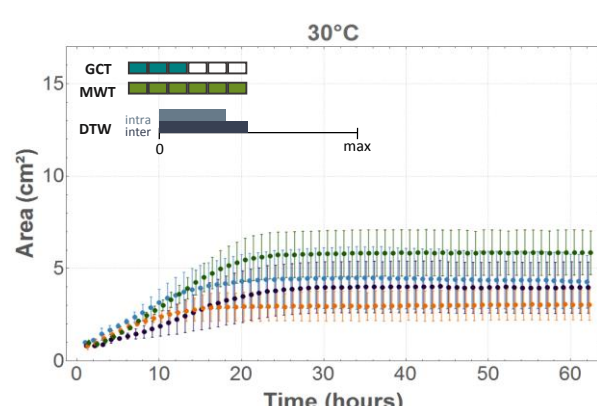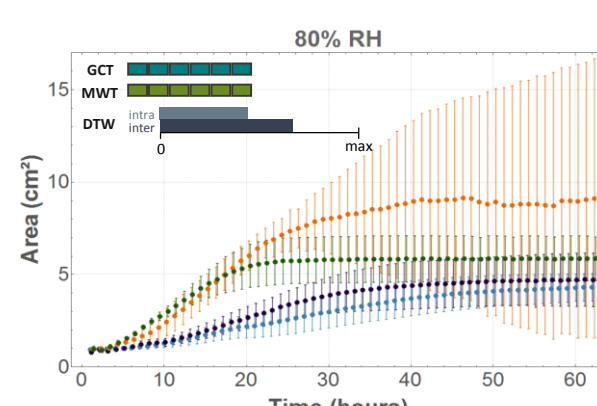

— 65%RH  
— 70%RH  
— 75%RH  
— 80%RH

— 15°C  
— 20°C  
— 25°C  
— 30°C

Supplement: Supplementary file 3 — Figure S3. Evolution of mycelial area (cm2) over time for R. solani. (PDF 604 kb) [file 43008_2019_9_MOESM3_ESM.pdf]

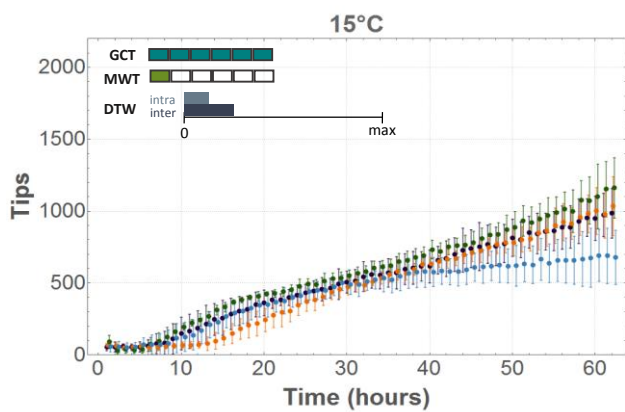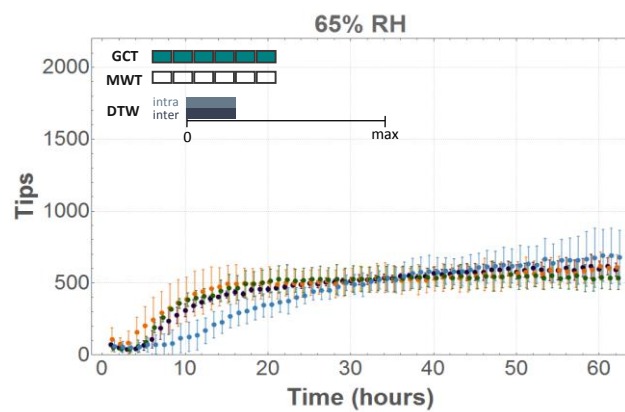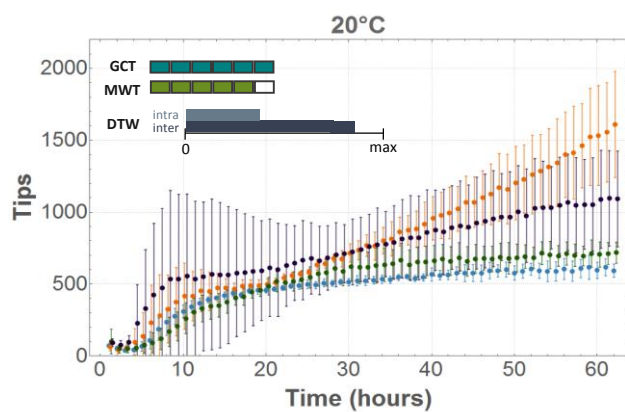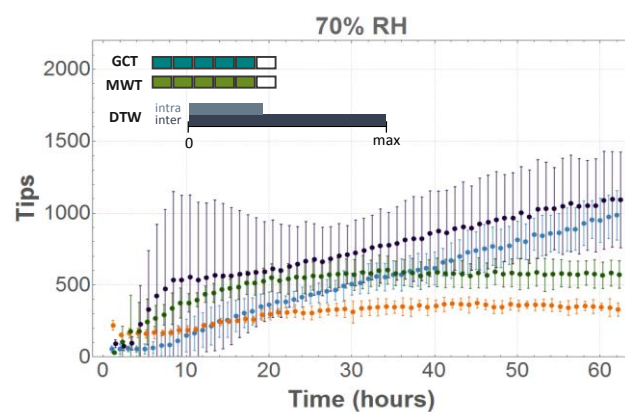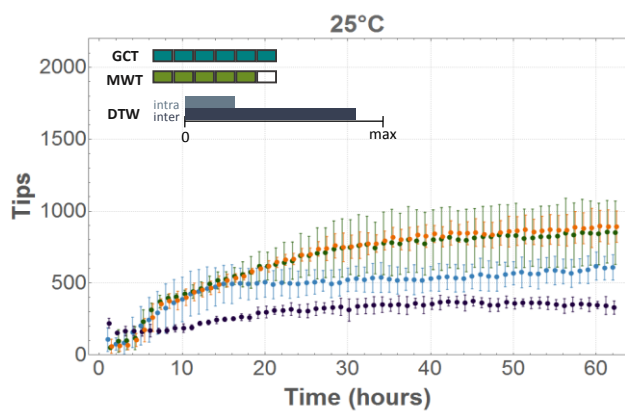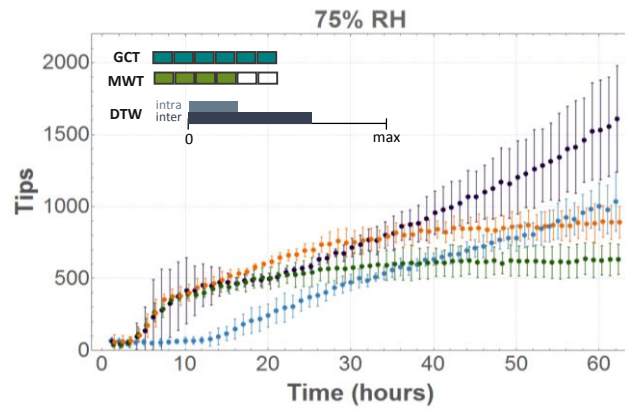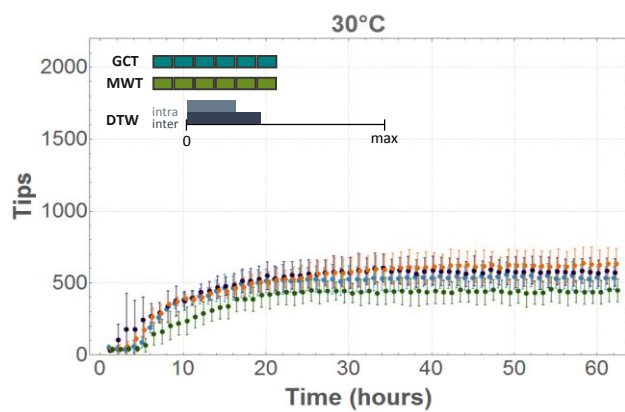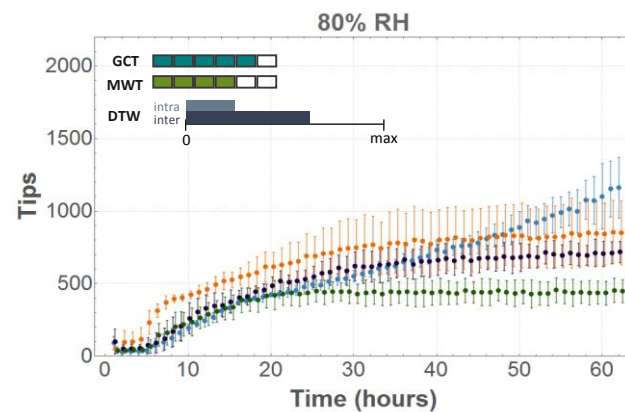

— 65%RH  
— 70%RH  
— 75%RH  
— 80%RH

— 15°C  
— 20°C  
— 25°C  
— 30°C

Supplement: Supplementary file 4 — Figure S4. Evolution of the number of tips over time for C. puteana. (PDF 459 kb) [file 43008_2019_9_MOESM4_ESM.pdf]

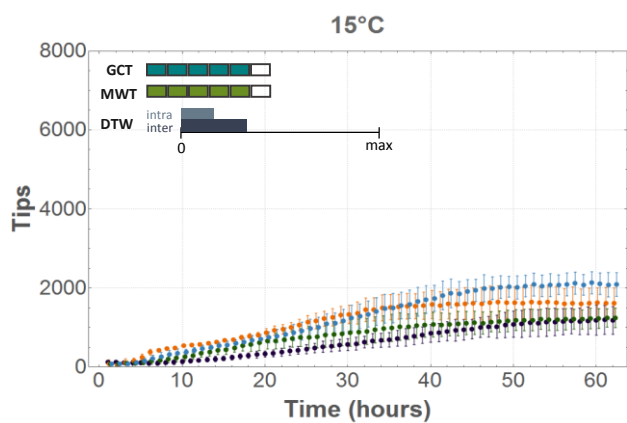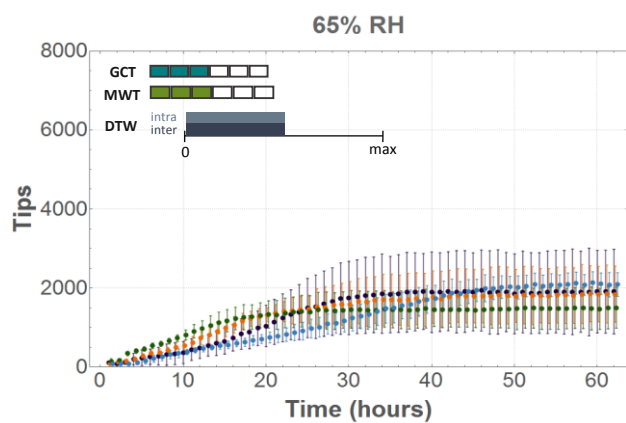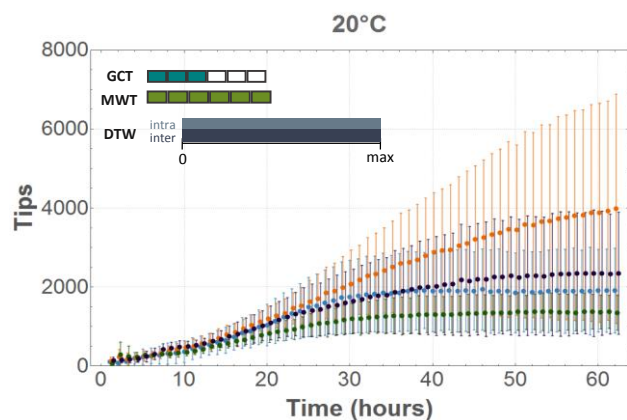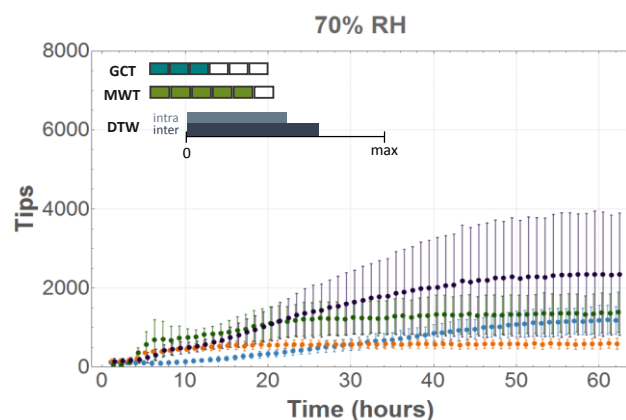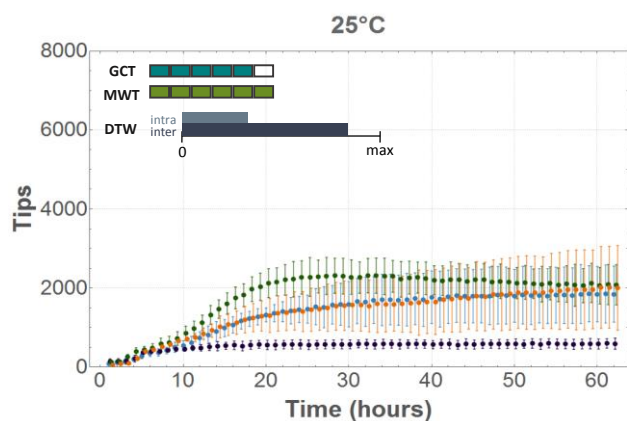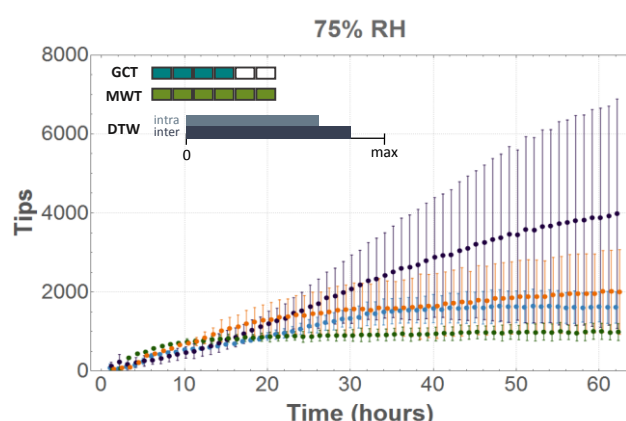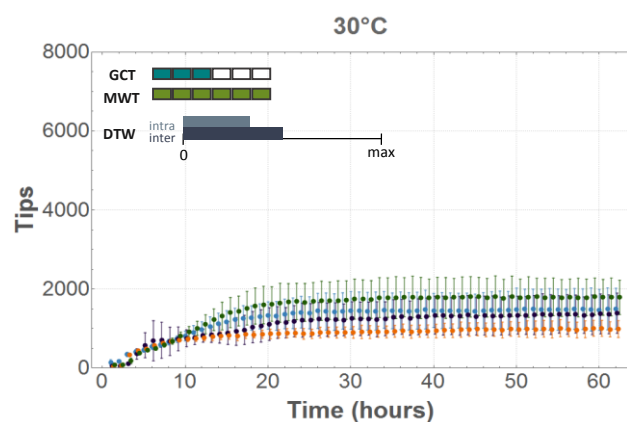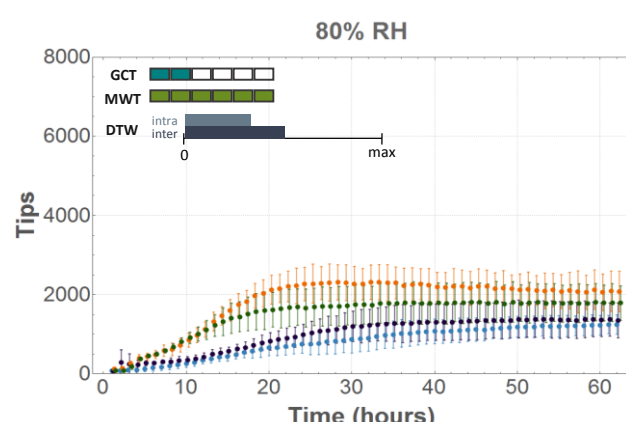

— 65%RH  
— 70%RH  
— 75%RH  
— 80%RH

— 15°C  
— 20°C  
— 25°C  
— 30°C

Supplement: Supplementary file 5 — Figure S5. Evolution of the number of tips over time for R. solani. (PDF 594 kb) [file 43008_2019_9_MOESM5_ESM.pdf]
